# High levels of domestic transmission and drug resistance –analysis of national surveillance data for shigellosis, the Netherlands, 2018 to 2024

**DOI:** 10.2807/1560-7917.ES.2026.31.41.2600075

**Published:** 2026-10-15

**Authors:** Boas van der Putten, Hendrik Sauskojus, Anouk Meijs, Daan Notermans, Maren Lanzl, Eelco Franz, Maaike van den Beld, Roan Pijnacker, on behalf of the ISIS-AR study group

**Affiliations:** 1 Centre for Infectious Disease Control, National Institute for Public Health and the Environment (RIVM), Bilthoven, Netherlands; 2 ECDC Fellowship Programme, Public Health Microbiology path (EUPHEM), European Centre for Disease Prevention and Control (ECDC), Stockholm, Sweden; 3 ECDC Fellowship Programme, Field Epidemiology path (EPIET), European Centre for Disease Prevention and Control (ECDC), Stockholm, Sweden; 4 The members of the ISIS-AR group are listed under Acknowledgements

**Keywords:** Shigellosis, antimicrobial resistance, surveillance, whole-genome sequencing, the Netherlands

## Abstract

**BACKGROUND:**

Antimicrobial resistance (AMR) among *Shigella* species is increasing, which is concerning. Strengthening surveillance is key to obtaining accurate, timely and actionable data on shigellosis.

**AIM:**

We aimed to describe Dutch national shigellosis surveillance data from 2018 to 2024, spanning the implementation of national genomic surveillance in 2022.

**METHODS:**

We described notified shigellosis cases and used logistic regression models to analyse associations between shigellosis and age, sex, year of infection, province, MSM (men who have sex with men) association, travel status, severity of disease and *Shigella* species.

**RESULTS:**

In 2018–2024, 3,020 shigellosis cases were notified. Incidence of shigellosis decreased during the COVID-19 pandemic years 2020–2021 but returned to pre-pandemic incidence in 2023 and 2024. Most cases were males (n = 1,887; 62.5%). Of the 1,731 cases with available data, 732 (42.3%) reported recent contact with MSM, representing 24.2% of all shigellosis cases. The proportion of extensive drug resistant (XDR) isolates increased from 2018 (1/58) to 2023 (24/109; 22.0%) (p < 0.001). Cases were more likely to be part of a cluster if they reported a recent MSM contact (p < 0.001) or harboured an XDR isolate (p < 0.001). Cases with recent MSM contact were more likely to harbour an XDR strain (p < 0.001).

**CONCLUSION:**

Implementation of genomic surveillance facilitated detection of clusters, provided more timely data on AMR and allowed for more detailed analyses of risk factors for *Shigella* transmission. Continued intervention efforts promoting hygiene and raising awareness on shigellosis and AMR are important to curb the increase of XDR shigellosis.

**Key public health message**

**What did you want to address in this study and why?**

*Shigella* bacteria can cause an intestinal infection (shigellosis) with severe diarrhoea and stomach cramps. In the Netherlands, approximately 400–600 persons are annually diagnosed with shigellosis. Antibiotics are becoming less effective against shigellosis. We wanted to investigate shigellosis in the Dutch population between 2018 and 2024.

**What have we learnt from this study?**

Between 2018 and 2024, 3,020 shigellosis cases were notified. In recent years, shigellosis cases often reported international travel or sexual contact between men. Almost half of the cases were infected in the Netherlands. As reported in other countries, antibiotics are becoming less effective against *Shigella* infections in the Netherlands. We were able to track transmission of *Shigella* bacteria better with genomic data.

**What are the implications of your findings for public health?**

Our findings highlight the ongoing need for promoting hygiene and of raising awareness about antimicrobial resistance, especially among travellers and men who have sex with men. Continued public health messaging on symptoms recognition and prevention is crucial for these risk groups.

## Introduction

Shigellosis is an intestinal infection caused by *Shigella* spp. This illness typically manifests with acute watery diarrhoea, fever, vomiting or abdominal cramps, but can lead to severe intestinal complications [1]. *Shigella* spp. are transmitted through faecal-oral contact and a very low infectious dose of the bacteria can cause an infection. Prevention mainly consists of good sanitary conditions and common hygienic measures. Antimicrobials are reserved for the treatment of severe infections, as mild cases typically recover within a week. Due to growing antimicrobial resistance (AMR), *Shigella* spp. are classified as high-priority pathogens by the World Health Organization (WHO) [2].

Annually, over 200 million shigellosis infections and over 200,000 deaths are estimated to occur worldwide [3]. The disease can be endemic in high-income countries, where imported and domestically acquired shigellosis pose a substantial public health burden, with growing concerns around extensive drug resistance (XDR) [4]. In many countries, shigellosis is also often associated with transmission among men who have sex with men (MSM) [1].

In the Netherlands, 400–600 shigellosis cases are annually reported to the National Institute for Public Health and the Environment (RIVM). Previously it was estimated that only one in 53 cases was detected, corresponding to an estimated true number of 27,000 shigellosis cases per year in the Netherlands [5].

The most recent update on national shigellosis surveillance data in the Netherlands covered data up to 2017 [6]. Since then, whole genome sequencing (WGS) surveillance has been implemented in the Netherlands and multiple international reports have been published on the rise of AMR in *Shigella* spp. [7]. We aimed to investigate epidemiology of shigellosis in the Netherlands from 2018 to 2024, including genomic epidemiology, explore risk factors for transmission and analyse AMR.

## Methods

### Epidemiological surveillance data and setting

Data on shigellosis cases were obtained from the registration system for notifiable infectious diseases (OSIRIS) at the RIVM. Shigellosis is notifiable for any person with (i) a positive culture for *Shigella* spp. or (ii) at least one of the four symptoms fever, diarrhoea, vomiting or abdominal pain and recent (< 2 weeks) contact with a culture-confirmed case [8]. Detection of *Shigella* spp. by PCR alone is not notifiable as accurate differentiation between *Shigella* spp. and enteroinvasive *Escherichia coli* still requires culture. Cases are notified by a physician or the medical microbiological laboratory (MML) to the municipal health service which conducts source and contact tracing with a standardised questionnaire (including the question “Did you have MSM contact in the week before illness onset?”). Pseudonymised information on disease manifestation, demographics (age, postal code, sex) and risk factors, such as travel history, is shared with the RIVM. Domestic and non-domestic transmission is based on the most likely country of infection, as ascertained through the questionnaire.

### Genomic surveillance data

Since 1 May 2022, all MMLs in the Netherlands have been asked to voluntarily submit *Shigella* spp. isolates for national genomic surveillance. We included all genomic *Shigella* data from the national genomic surveillance that could be linked to a notified case. The isolates were whole genome sequenced using Illumina NextSeq (Illumina, San Diego, the United States (US)) and subsequently analysed with Juno pipelines (https://github.com/RIVM-bioinformatics). Mykrobe version 0.13.0 (https://bioconda.github.io/recipes/mykrobe/README.html) and sonneityping version 20210201 (https://github.com/ShigellaGenomics/sonneityping) were used to assign *S. sonnei* lineages. Clustering analysis was carried out using Ridom SeqSphere (https://www.ridom.de/seqsphere/ug/v40/Overview.html), including allele calling.

Genetic clusters were defined based on core genome multilocus sequence typing (cgMLST), using the Enterobase *Escherichia*/*Shigella* scheme version 1 [9]. Clusters were assigned using single-linkage clustering with a threshold of ≤ 5 alleles difference.

### Antimicrobial susceptibility testing data

Antimicrobial susceptibility testing data for isolates sampled from 2018 to 2023 were collected from the Dutch Infectious Diseases Surveillance Information System for Antimicrobial Resistance (ISIS-AR) which is based on medical routine diagnostics [10]. Data for cefotaxime/ceftriaxone, ceftazidime, ciprofloxacin, co-trimoxazole and meropenem/imipenem were selected for analysis. Subsequently, isolates were categorised as susceptible (including susceptible, increased dosing) or resistant by reinterpretation of minimum inhibitory concentrations (MIC) and inhibition zone diameters according to European Committee on Antimicrobial Susceptibility Testing (EUCAST) clinical breakpoints version 14.0 (https://www.eucast.org/bacteria/clinical-breakpoints-and-interpretation/clinical-breakpoint-tables/).

### Data linkage

Case notifications were linked to the genomic surveillance data and AST data using score-based matching with following matching criteria: age, date of onset (or if unavailable date of sampling), postal code (four digits) and sex.

### Definition of extensive drug resistance

Isolates were considered XDR if they either harboured genetic resistance determinants or displayed phenotypic resistance against all four types of antimicrobials recommended by the Dutch guidelines [11]. The four antimicrobials we included were, with the relevant genetic resistance determinants written in parentheses: (i) cefotaxime/ceftriaxone and/or ceftazidime (any ESBL or AmpC gene); (ii) ciprofloxacin (at least two of *gyrA* S83L, *gyrA* D87N/G/Y, *parC* S80I/R or *qnrS*1); (iii) co-trimoxazole (one of *sul*1, *sul*2 or *sul*3 and one of *dfrA*1, *dfrA*14 or *dfrA*17) and (iv) azithromycin (one of *mphA*, *ermB*). If data were missing for one or more relevant antimicrobials, isolates could not be classified as XDR.

Annual proportions of phenotypically and genotypically predicted resistant isolates were compared to assess concordance between methods at the population level.

### Statistical analysis

All statistical analyses were performed using R version 4.4.0 (https://www.r-project.org/). We included cases from 1 January 2018 to 31 December 2024 and genomic data from 1 May 2022 to 31 December 2024. Cases without an identification code (ID) or with a duplicated ID were excluded.

Determination of MSM association was based on self-reported MSM contact in the week prior to the onset of symptoms. A binary cluster variable was created for clusters with at least two related cases.

Cases were described by age, sex, having MSM contact in the week before illness onset (categorised as yes, no or unknown), country of infection, severity of disease (requiring hospitalisation) and *Shigella* species. The annual incidence of shigellosis per 100,000 population was calculated at the provincial and national level. Representativeness of genomic surveillance was assessed by comparing cases with and without linked genome sequences on age, sex, year, province, MSM association, travel status, severity of disease and *Shigella* species using multivariable logistic regression.

We used chi-square test to compare categorical variables and a two-proportion z-test for comparing proportions. A two-tailed p value of less than 0.05 was considered statistically significant. Combinations of two variables with associations in cross-tabulations and previous studies on shigellosis were assessed using bivariate logistic regression. As a result, we investigated sex, MSM association and travel history as exposures, and their associations with being part of a cluster, the severity of the infection, having an XDR strain and travel history as outcomes.

Considering the results from the univariate regression models and relationships between two variables that seemed to be confounded by other variables resulted in the construction of two multivariable logistic regression models. As being part of a cluster satisfied the conditions for a potential confounder of the relationship between having an MSM association (exposure) and travel history (outcome), this was tested in the first model. The second model, based on the same assumptions of confounding, examined the association between MSM association (exposure) and being part of a cluster (outcome), adjusted for being a domestic or non-domestic case. Multicollinearity was assessed using variance inflation factor analysis.

Testing statistical significance of changing resistance proportions over time was done by using univariate logistic regression of resistance to azithromycin, ciprofloxacin, co-trimoxazole and third-generation cephalosporins, using year as continuous independent variable and Benjamini–Hochberg correction for multiple testing.

## Results

We included 3,020 notified cases of shigellosis in the analysis after excluding cases with missing IDs (n = 72). Of the notified cases, 1,133 (37.5%) were females and 1,887 (62.5%) were males. Female cases were a median age of 37 years (interquartile rage (IQR): 25–54), while male cases were a median age of 41 years (IQR: 29–55). Of the 3,020 notified cases, 105 (3.5%) were not laboratory-confirmed. The incidence was highest in the province of Noord-Holland, which includes Amsterdam (Figure 1).

**Figure 1:**
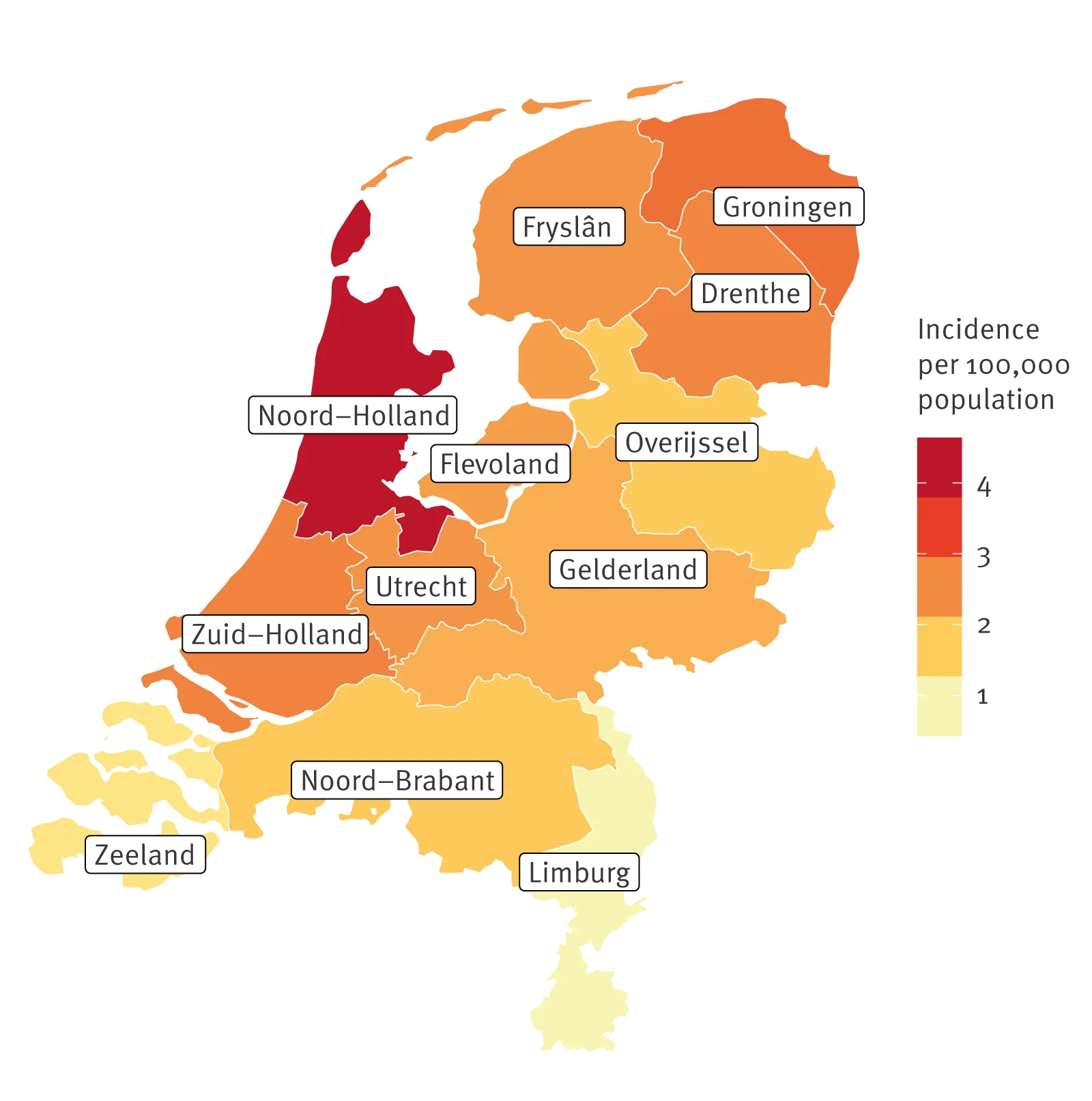
Average incidence of shigellosis per 100,000 population, by province, the Netherlands, 2018–2024 (n = 3,020)

In 2018, 3.01 cases per 100,000 population (n = 518) were notified, dropping to 1.13 and 1.26 cases per 100,000 population in 2020 (n = 196) and 2021 (n = 221), respectively. The incidence increased between 2022 and 2023, with 2.50 (n = 440) and 3.39 (n = 604) cases per 100,000 population, respectively, but decreased to 2.73 cases per 100,000 population in 2024 (n = 490) (Figure 2). On average, 46.2% of cases were domestic, with the highest proportions in 2020 (70.7%) and 2021 (70.4%) and the lowest in 2018 (36.4%).

**Figure 2:**
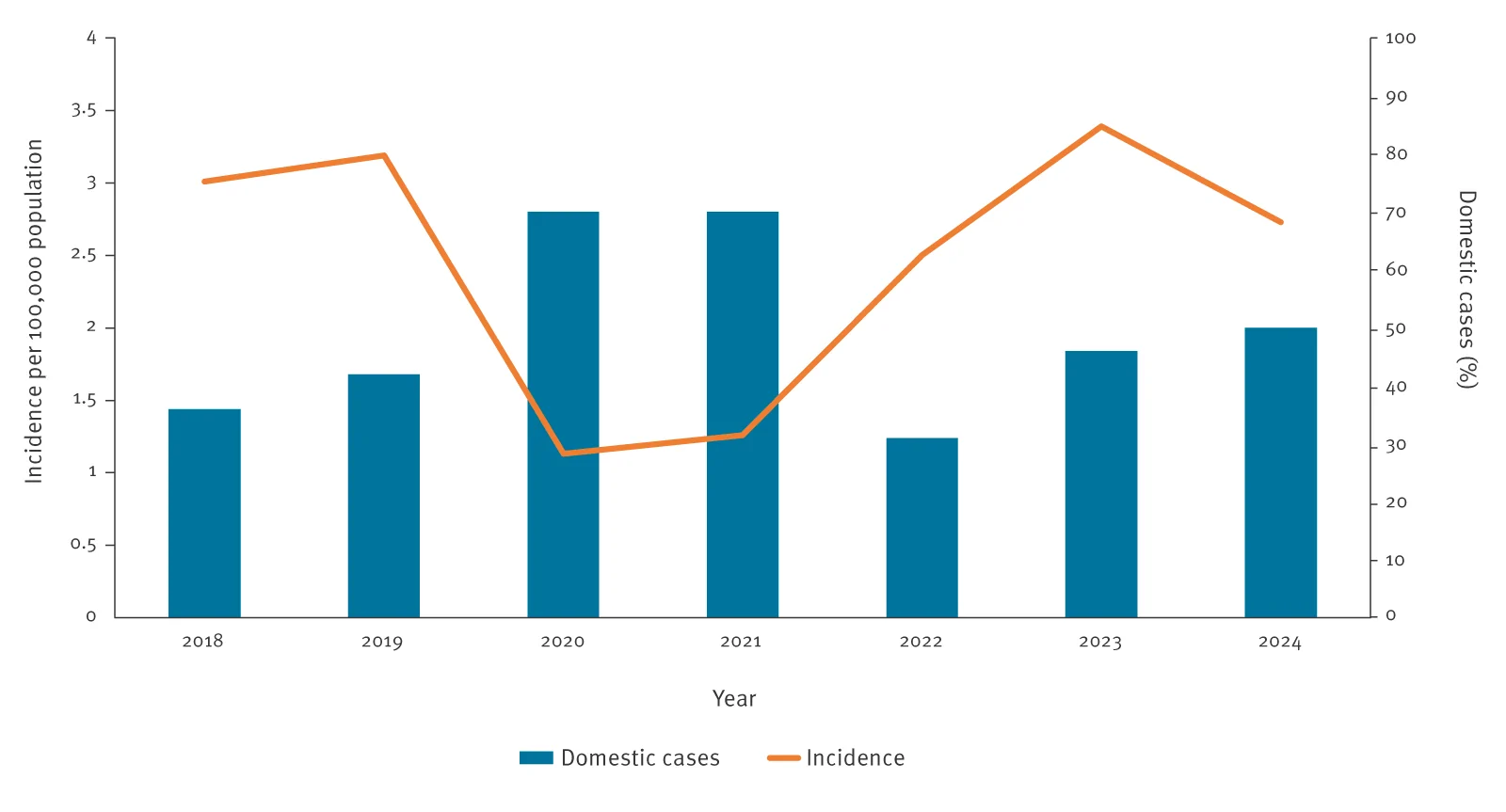
Incidence of shigellosis per 100,000 population and percentage of domestic cases, the Netherlands, 2018–2024 (n = 3,020)

Most notified cases were males, with a high proportion of 72.9% (161/221) in 2021, and in those aged 21–50 years (n = 1,665; 55.1%), followed by those aged ≥ 51 years (n = 1,046; 34.6%) (Table 1). Of the 1,731 cases with available data, 732 (42.3%) reported recent MSM contact, representing 24.2% of all shigellosis cases. The proportion of MSM-associated cases among males increased from 31.2% in 2018 to 41.6% in 2024, with a peak of 48.6% in 2020. Among MSM-associated cases, 82.1% (n = 601) were considered domestically acquired, compared with 43.3% (n = 433) of male cases that reported no MSM contact.

**Table 1:** Characteristics of shigellosis cases, the Netherlands, 2018–2024 (n = 3,020)

Characteristic	2018 (n = 518)		2019 (n = 551)		2020 (n = 196)		2021 (n = 221)		2022 (n = 440)		2023 (n = 604)		2024 (n = 490)		Total (n = 3,020)	
	n	%	n	%	n	%	n	%	n	%	n	%	n	%	n	%
Sex																
Female	239	46.1	206	37.4	54	27.6	60	27.1	181	41.0	225	37.3	168	34.3	1,133	37.5
Male	279	53.9	345	62.6	142	72.4	161	72.9	259	59.0	379	62.7	322	65.7	1,887	62.5
Age (years)																
0–5	16	3.1	18	3.3	2	1.0	10	4.5	14	3.2	15	2.5	16	3.3	91	3.0
6–10	26	5.0	20	3.6	2	1.0	6	2.7	15	3.4	19	3.1	12	2.4	100	3.3
11–20	24	4.6	23	4.3	9	4.6	11	5.0	15	3.4	25	4.1	13	2.7	120	4.0
21–50	279	53.9	295	53.5	122	62.2	137	62.0	235	53.4	334	55.3	261	53.3	1,663	55.1
≥ 51	173	33.4	195	35.4	61	31.1	57	25.8	161	36.6	211	34.9	188	38.4	1,046	34.6
Men who have sex with men																
Yes	87	31.2	147	42.6	69	48.6	73	45.3	86	33.2	139	36.7	131	40.7	732	38.8
No	169	60.6	173	50.1	65	45.8	75	46.6	152	58.7	211	55.7	154	47.8	999	52.9
Unknown	23	8.2	25	7.2	8	5.6	13	8.1	21	8.1	29	7.7	37	11.5	156	8.3
*Shigella* species (n = 2,958)																
*S. sonnei*	316	62.8	347	64.0	85	43.6	96	44.0	273	64.1	423	70.7	256	53.8	1,796	60.7
*S. flexneri*	155	30.8	155	28.6	100	51.3	111	50.9	123	28.9	161	26.9	192	40.3	997	33.7
*S. boydii*	17	3.4	17	3.1	9	4.6	9	4.1	16	3.8	9	1.5	18	3.8	95	3.2
*S. dysenteriae*	15	3.0	23	4.2	1	0.5	2	0.9	14	3.3	5	0.8	10	2.1	70	2.4
Travelled outside the Netherlands (n = 1,568)																
Female	189	59.1	173	55.4	31	55.4	28	44.4	142	53.0	173	54.2	118	51.3	854	54.5
Male	131	40.9	139	44.6	25	44.6	35	55.6	126	47.0	146	45.8	112	48.7	714	45.5

Among cases with information on *Shigella* species (n = 2,958), most were caused by *S. sonnei* (n = 1,796; 60.7%) or *S. flexneri* (n = 997; 33.7%), whereas *S. boydii* (n = 95; 3.2%) and *S. dysenteriae* (n = 70; 2.4%) were much less common. Most *S. sonnei* infections with information on the country of transmission (n = 1,752) were contracted in the Netherlands (n = 739; 42.2%), followed by Morocco (n = 36; 2.1%). Similarly, most cases of *S. flexneri* were contracted in the Netherlands (552/947; 58.2%), followed by Morocco (140/947; 14.8%). In 2020–2021, the proportion of cases caused by *S. flexneri* increased in the Netherlands compared with 2018–2019.

The two most common countries among travel-related cases were Morocco (n = 194; 12.4%) and Egypt (n = 169; 10.8%). In 2022, most travel-related cases came from Cabo Verde (n = 56; 12.7% of all cases in 2022), the centre of a multi-country outbreak of shigellosis [12]. Female cases were more likely to report recent travel and also contracted shigellosis abroad more often than male cases, such as Morocco (n = 115; 59.3%) and Egypt (n = 110; 65.1%). However, notable exceptions were Spain (male cases: 52/59) and Germany (male cases: 34/36), where a higher proportion of male cases were observed. Spain (n = 36) and Germany (n = 28) were also the most common countries among 125 non-domestic cases with an MSM association.

### Severity of disease

A higher proportion of male cases (17.9%; n = 338) was admitted to a hospital compared with female cases (11.9%; n = 135) (p < 0.001). The authors make an additional table of comparisons by sex and recent MSM contact available in Supplementary Table 1. There was no significant difference in hospitalisation between MSM-associated and non-MSM-associated male cases (p = 0.3). Two hundred forty-one (13.4%) *S. sonnei* cases and 211 (21.2%) *S. flexneri* cases were hospitalised*.*

### Representativeness of genomic surveillance

Since the start of the comprehensive genomic surveillance in 2022, 50.6% (776/1,534) of notified cases were matched to genomic data, with isolates submitted by nearly all MMLs in the Netherlands (n = 43). Only cases with *S. boydii* (n = 39) (OR = 0.65; 95% confidence interval (CI): 0.50–0.84; p < 0.001) or *S. dysenteriae* (n = 27) (OR = 0.58; 95% CI: 0.41–0.81; p = 0.001) infections were less likely to have a linked genome sequence, compared with cases with a *S. flexneri* infection (n = 446) since the start of comprehensive genomic surveillance. Additionally, the proportion of cases linked to a genome sequence differed between provinces (p < 0.001). For one province (Fryslân), no cases could be linked to genome sequences (0/49), while genome linkage among cases ranged from 15 of 54 to 100 of 140 for other provinces. No significant association could be identified between linkage of a genome sequence with either transmission mode (domestic vs. travel-acquired), recent MSM contact, age, year, antimicrobial resistance or hospitalisation when adjusting for all investigated variables.

### Trends in phenotypic and genotypic antimicrobial resistance

Some antimicrobial susceptibility data were available from 2,089 cases: 1,313 with only phenotypic data, 347 with only genomic data and 429 with both phenotypic and genomic data. The proportion of isolates resistant against third-generation cephalosporins increased from 15.3% (49/321) in 2018 to 40.3% (137/340) in 2023 (p < 0.001). Ciprofloxacin resistance among isolates increased from 26.3% (92/350) to 39.4% (156/396) (p = 0.041). The proportion of XDR isolates increased from 1.7% (1/58) in 2018 to 22.0% (24/109) in 2023 (p < 0.001), based on isolates with phenotypic data available for all four antimicrobials. No statistically significant changes were observed for co-trimoxazole (2018: 254/350; 72.6% and 2023: 277/391; 70.8%) and azithromycin (2018: 22/65; 33.8% and 2023: 46/134; 34.3%). No isolates showed carbapenem resistance or harboured carbapenemase genes. The authors make additional visualisations available in Supplementary Figures 1 and 2, describing antimicrobial resistance by species and transmission (domestically acquired versus travel-acquired).

Phenotypic AST data for all four antimicrobials or antimicrobial groups were reported for only 14.5% (439/3,020) of cases, particularly due to the low completeness of azithromycin data (16.4%; 495/3,020). An additional overview of the completeness of antimicrobial susceptibility data is available in Supplementary Table 2. For ceftazidime, ciprofloxacin and co-trimoxazole, around 80% of AST data originated from automated testing systems, while nearly all azithromycin AST data originated from E-tests. For cefotaxime/ceftriaxone, this information was not readily available.

Presence of genotypic resistance markers corresponded well to phenotypic AMR on a population level (Figure 3). A total of 153 cases with an XDR isolate were identified, of which 112 solely in genomic surveillance and 21 solely in phenotypic AST surveillance. The remaining 20 XDR cases were identified in both genomic and phenotypic surveillance. Either genotypic or phenotypic susceptibility data on all four relevant antimicrobials was available for 1,100 cases.

**Figure 3:**
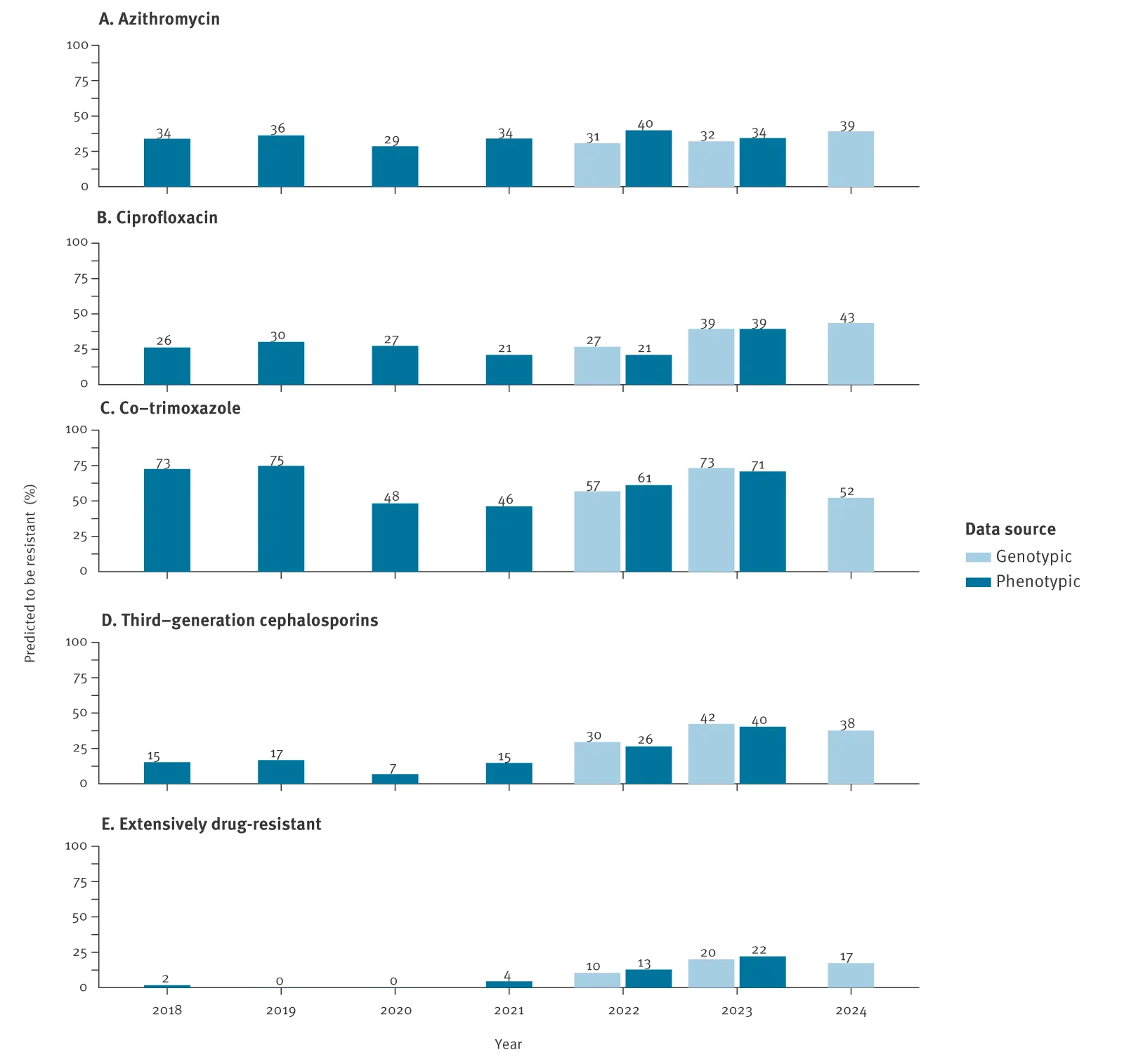
Percentages of *Shigella* species isolates either genotypically predicted to be resistant or phenotypically resistant against azithromycin, ciprofloxacin, co-trimoxazole and third-generation cephalosporins, the Netherlands, 2018–2024 (n = 2,089)^a^

^a^ Only phenotypic data (azithromycin: n = 378; ciprofloxacin: n = 1,304; co-trimoxazole: n = 1,300; third-generation cephalosporins: n = 1,197; extensive drug resistance: n = 1,265); only genomic data (azithromycin: n = 659; ciprofloxacin: n = 347; co-trimoxazole: n = 351; third-generation cephalosporins: n = 394; extensive drug resistance: n = 392), phenotypic and genomic data (azithromycin: n = 117; ciprofloxacin: n = 429; co-trimoxazole: n = 425; third-generation cephalosporins: n = 382; extensive drug resistance: n = 384).

### Description of detected genetic clusters

Nine genetic clusters with ≥ 20 cases of *S. sonnei* (n = 6) and *S. flexneri* (n = 3) were identified in genomic surveillance between 2022 and 2024 (Table 2). Among these clusters, cases reported either predominantly recent MSM contact (six clusters) or recent international travel (three clusters). Three of the *S. sonnei* clusters in which cases reported MSM contact comprised XDR isolates. Clusters 1 and 7 mostly comprised XDR isolates, accounting for 58.2% (89/153) of all XDR cases. Both clusters (1 and 7) were reported in other countries [13-24] and belonged to lineages 3.6.1.1 and 3.6.1.1.2. Cluster 1 harboured *bla*_CTX-M-15_ as ESBL gene while cluster 7 harboured *bla*_CTX-M-27_. Thirteen of 44 isolates in Cluster 2 were XDR and belonged to *S. sonnei* lineage 3.6.1.1. The first Dutch case of this cluster was detected in November 2022, and in June 2024, cases with XDR were identified. This cluster gained XDR through the acquisition of *bla*_DHA-1_ and *mphA*. An additional epidemiological curve of this cluster is available in Supplementary Figure 3.

**Table 2:** Description of the nine largest genetic clusters of *Shigella* species identified in genomic surveillance, the Netherlands, May 2022–2024^a^

Cluster	Species (lineage)	Cases (n)	MSM		Travel history		Country of travel	Antimicrobial	XDR	Relation to previous studies	Enterobase HC5 label
			n	%	n	%					
1	*S. sonnei* (3.6.1.1)	72	44	61.1	13	18.1	Several countries	3GC, AZM, CIP, TMP-SMX	Yes	[14-16]	194512
2	*S. sonnei* (3.6.1.1)	44	24	54.5	10	22.7	Several countries	3GC^b^, AZM^b^, CIP, TMP-SMX	Yes^b^	No studies	219392
3	*S. sonnei* (3.6.2)	41	0	0	28	68.3	Morocco	TMP-SMX	No	[13]	26701
4	*S. sonnei* (3.7.17)	34	0	0	30	88.2	Cabo Verde	TMP-SMX^c^	No	No studies	181425
5	*S. flexneri*	29	16	55.2	4	13.8	Several countries	3GC, CIP	No	[17,32]	181248
6	*S. flexneri*	25	16	64.0	2	8.0		ND^d^	No	[13,18,19,33]	81361
7	*S. sonnei* (3.6.1.1.2)	24	16	66.7	6	25.0		3GC, AZM, CIP, TMP-SMX	Yes	[13,17-24]	98748
8	*S. sonnei* (3.7.25)	22	0	0	16	72.7	Jordan, Egypt	3GC, TMP-SMX	No	[14,24,34]	127002
9	*S. flexneri*	20	16	80.0	4	20.0	Several countries	AZM, CIP	No	[17]	189644

3GC: third generation cephalosporins; AZM: azithromycin; CIP: ciprofloxacin; HC5: hierarchical cluster at 5 allelic differences; MSM: men who have sex with men; ND: not detected; TMP-SMX: co-trimoxazole; XDR: extensive drug resistance.

^a^ Percentages are calculated from the number of cases with available data for that variable. Predominant country of travel is indicated only for clusters where most cases reported recent travel outside the Netherlands.

^b^ A sub-cluster of 13 cases with isolates typed as XDR due to additional resistance against third-generation cephalosporins and azithromycin.

^c^ 17 cases with isolates encoding co-trimoxazole resistance.

^d^ Resistance markers against first line antimicrobials were not detected.

### Regression analysis

#### Univariate logistic regression

Male cases had higher odds of being hospitalised (OR = 1.62; 95% CI: 1.31–2.02; p < 0.001) but were less likely to be a non-domestic case when compared with female cases (OR = 0.19; 95% CI: 0.16–0.23; p < 0.001) (Table 3). Male cases also had higher odds of having been infected with an XDR strain (OR = 4.22; 95% CI: 2.69–6.99; p < 0.001) and being part of a cluster (OR = 2.16; 95% CI: 1.58–2.95; p < 0.001).

**Table 3:** Associations between variables associated with shigellosis in univariate regression analysis, the Netherlands, 2018–2024 (n = 3,020)

Variable	Hospitalisation			Being part of a cluster			XDR strain			Travel outside the Netherlands		
	Estimate	95% CI	p value	Estimate	95% CI	p value	Estimate	95% CI	p value	Estimate	95% CI	p value
Sex (reference: female)												
Male	1.62	1.31–2.02	< 0.001	2.16	1.58–2.95	< 0.001	4.22	2.69–6.99	< 0.001	0.19	0.16–0.23	< 0.001
MSM association (reference: no)												
MSM contact	0.86	0.67–1.11	0.24	3.14	1.96–5.15	< 0.001	3.15	2.15–4.69	< 0.001	0.17	0.13–0.21	< 0.001
History of travel (reference: no)												
Travelled	0.41	0.36–0.51	< 0.001	0.30	0.21–0.41	< 0.001	0.35	0.24–0.50	< 0.001	NA		

CI: confidence interval; MSM: men who have sex with men; NA: not applicable; XDR: extensive drug resistance.

Cases with an MSM association were not more likely to be hospitalised (OR = 0.86; 95% CI: 0.66–1.11; p = 0.24), but had higher odds of being part of a *Shigella* cluster (OR = 3.14; 95% CI: 1.96–5.15; p < 0.001), harbouring an XDR strain (OR = 3.15; 95% CI: 2.15–4.69; p < 0.001) and lower odds of having acquired the infection outside the Netherlands (OR = 0.17; 95% CI: 0.13–0.21; p < 0.001) when compared with male cases without an MSM association.

Non-domestic cases had lower odds of belonging to a *Shigella* cluster (OR = 0.30; 95% 0.21–0.41; p < 0.001) and being hospitalised (OR = 0.41; 95% CI: 0.36–0.51; p < 0.001). Lastly, cases with XDR were more likely to be part of a cluster (OR = 4.52; 95% CI: 2.66–8.27; p < 0.001), but not more likely to be hospitalised.

### Multinomial logistic regression

Transferring the relationship between having an MSM association (predictor) and being a non-domestic case (outcome) into a model that adjusted for clustering, the initial OR (0.17; 95% CI: 0.13–0.21) increased to 0.23 (95% CI: 0.15–0.37). The association between having an MSM association (predictor) and being part of a cluster (outcome) (OR = 3.14; 95% CI: 1.96–5.15) weakened when adjusted for the domestic or non-domestic transmission (OR = 2.23; 95% CI: 1.33–3.81).

Including the year of infection, age or *Shigella* species as a covariate did not significantly alter the relationship between being a non-domestic case and MSM association or being part of a cluster and MSM association.

## Discussion

The incidence of shigellosis in the Netherlands varied significantly across the years 2018–2024, with a marked decline during the COVID-19 pandemic years of 2020–2021, likely due to a decrease in travel-related cases, similar to other gastrointestinal infections [25]. In 2020–2021, most cases in the Netherlands were caused by *S. flexneri* which contrasts with the European Centre for Disease Prevention and Control (ECDC) reporting more *S. sonnei* than *S. flexneri* cases during this period across the European Union/European Economic Area (EU/EEA) countries [26,27]. Males accounted for a higher proportion of cases, largely driven by MSM-associated transmission, and had higher odds of being hospitalised and being part of a genomic cluster. Cases associated with MSM had higher odds of having acquired their infection domestically and harbouring an XDR *Shigella* strain, while XDR status was also associated with an increased odds of being part of a genomic cluster, but not with being hospitalised. This risk was lower in non-domestic cases compared with domestic ones. However, all logistic regression analyses were exploratory, and we do not claim that the identified risk factors are causal, due to limitations in constructing more complex models. Several large clusters linked to other international clusters were detected in the Netherlands, two of which mainly comprised XDR isolates. Similar to other countries, we observed an increase in AMR among shigellosis cases in the Netherlands [4].

The incidence of shigellosis cases per 100,000 population was 30-40% higher in the Netherlands than in most other EU/EEA countries, for which public data are available up to 2022 [4]. Only a few countries, including Spain and France, showed higher shigellosis incidence rates. Germany, which was frequently mentioned in the travel histories of our cases, recorded a much lower incidence than the Netherlands. Differences between country incidence rates might be due to several reasons, including applied case definitions, coverage of the surveillance system and differences in healthcare systems.

In line with our findings, increased transmission among MSM is thought to contribute to the differences between case numbers among females and males in the other EU/EEA countries [4]. The proportion of MSM-associated cases among males in our analysis (42.2% in 2024) contrasts sharply with the estimated 6.9% of males in the Netherlands identifying as either gay or other gender and sexual minorities. In addition, some provinces, such as Noord-Holland, which include highly urbanised hubs where people from gender and sexual minority communities are more likely to live, such as Amsterdam [28], had the highest case numbers. These findings confirm that MSM contact is a risk factor for contracting shigellosis, as supported by other studies [4,29], and should hence continue to be addressed in tailored risk communication strategies, such as through targeted websites.

Both ECDC and WHO have identified the increasing AMR of *Shigella* as a public health concern [2,4]. This is in line with Dutch surveillance data which also showed an increase in phenotypic resistance against ciprofloxacin and third-generation cephalosporins in recent years. In genomic surveillance, 10-20% of cases in recent years were classified as XDR. Given the association between being part of a cluster and XDR status, it seems likely that expansion of resistant clones contributed to rising resistance.

Since 1 May 2022, all *Shigella* spp. isolates submitted to the RIVM are whole genome sequenced [13]. The implementation of genomic surveillance enabled detection of 61 clusters (with at least two cases), of which nine clusters comprised at least 20 cases. Many other high-income countries currently use routine WGS in surveillance [22,29], allowing comparison of international data and improved detection of cross-border transmission [4]. Seven of the nine large clusters in our study were also reported in other countries. Notably, five international clusters included only few cases with recent travel reported, suggesting predominantly domestic transmission despite being reported in multiple countries.

Additionally, routine WGS has enabled genotypic AMR prediction which is used in risk assessments of newly detected clusters. This allowed the detection of XDR emergence in a cluster which has been present in the Netherlands for at least 1.5 years. Genomic AMR surveillance complements phenotypic AMR surveillance well: results become available quicker at the national institute and genomic surveillance covers more resistance classes than usually tested by MMLs (e.g. for azithromycin). National genomic AMR surveillance can also inform specialised guidelines for treatment of people with a risk of acquiring resistant strains.

In the current questionnaire for shigellosis cases, MSM status is defined as reporting MSM contact within the week prior to the onset of disease. The results are, therefore, only extendable to these individuals, and not to people that are part of the general MSM community. Furthermore, among non-domestic cases, we were unable to distinguish between travel for family visits and tourism, limiting our ability to develop targeted interventions to prevent infections acquired abroad. Although shigellosis is a notifiable disease, there is likely substantial underreporting [5], and cases in our study may represent more severe cases or more resistant infections [30]. Genomic data were more commonly available for cases infected by *S. sonnei* and *S. flexneri*, and for cases in certain provinces. While the paucity of genomic data on *S. boydii* and *S. dysenteriae* is not ideal, *S. sonnei* and *S. flexneri* are most relevant for public health in the Netherlands due to their much higher incidence. The varying geographical coverage of genomic surveillance could influence the exact estimates of our regression analyses, although general findings would remain valid since other important variables were not associated with availability of genomic data.

The phenotypic azithromycin susceptibility data were mainly based on E-tests. For *Salmonella* Typhi, interpretation of the E-test might be challenging [31]. This might also be the case for *Shigella* spp., which might explain rare discrepancies between phenotypic and genotypic data in our study.

## Conclusion

In the Netherlands, shigellosis mainly affects risk groups such as MSM and travellers. In recent years, AMR has sharply increased, limiting treatment options. The implementation of routine WGS has enabled tracking of *Shigella* clusters, improved timely detection of AMR and allowed further analyses of risk groups. Furthermore, sexual transmission of resistant *Shigella* should be closely monitored and hygiene promotion to prevent faecal-oral transmission during sexual activities is crucial. Lastly, the linkage between epidemiological and microbiological data should be improved and the efforts to reduce underreporting should be increased, such as raising awareness about the increase in AMR, and specifically XDR, both of which also remain crucial for the prevention and control of shigellosis.

## Data Availability

Whole genome sequencing data have been deposited in the European Nucleotide Archive under BioProject PRJEB106896 (https://www.ebi.ac.uk/ena/browser/home). Epidemiological data are available upon reasonable request by contacting the corresponding author.

## Supplementary Data

1116000 Supplementary Material
