# Supplementary material for: High levels of domestic transmission and drug resistance –analysis of national surveillance data for shigellosis, the Netherlands, 2018 to 2024
Source: Euro Surveill. 2026 Oct 15;31(41):2600075. doi: 10.2807/1560-7917.ES.2026.31.41.2600075 (PMC13675696; doi:10.2807/1560-7917.ES.2026.31.41.2600075)
Supplement: Supplementary file 1 [file 26-00075_VANDERPUTTEN_Supplement.pdf]

This supplementary material is hosted by Eurosurveillance as supporting information alongside the article "High levels of domestic transmission and drug resistance – an analysis of Dutch national surveillance data for shigellosis, 2018 – 2024", on behalf of the authors, who remain responsible for the accuracy and appropriateness of the content. The same standards for ethics, copyright, attributions and permissions as for the article apply. Supplements are not edited by Eurosurveillance and the journal is not responsible for the maintenance of any links or email addresses provided therein.

Table S1. Comparison by sex and men who have sex with men (MSM) contact in the week before symptom onset. XDR = extensive drug resistance.

	Women	Men	p-value*	Non-MSM	MSM	p-value*
**Total**	N = 1,113	N = 1,899	-	N = 999	N = 732	-
**Mean Age (years)**	38.8	41.6	<0.001	40.3	42.1	0.023
**Most common *Shigella* species**	*S. sonnei* (n=768, 70%)	*S. sonnei* (n=1,029, 55%)	<0.001	*S. sonnei* (572, 58%)	*S. sonnei* (369, 51.0%)	0.006
**Most common country of infection**	Netherlands (n=254, 22.0%)	Netherlands (n=1,098, 58.0%)	<0.001	Netherlands (435, 43.0%)	Netherlands (601, 82.0%)	<0.001
**XDR**	25 (2.2%)	138 (7.3%)	<0.001	40 (4.0%)	88 (12.0%)	<0.001
**Hospitalised**	135 (12.0%)	338 (18.0%)	<0.001	183 (19%)	119 (16%)	0.271

Table S2. Completeness of phenotypic AST data from ISIS-AR for 2018-2023. Completeness is expressed as the percentage of cases that has phenotypic AST data available for that antimicrobial for that year.

Year	Azithromycin	Ciprofloxacin	Cefotaxime/ ceftriaxone/ ceftazidime	Co-trimoxazole
2018	12.5	67.6	66.6	67.6
2019	18.5	65.0	64.2	64.8
2020	21.4	70.9	70.4	70.9
2021	22.6	68.8	68.3	68.8
2022	22.3	73.6	71.6	73.2
2023	22.2	65.6	63.2	64.7

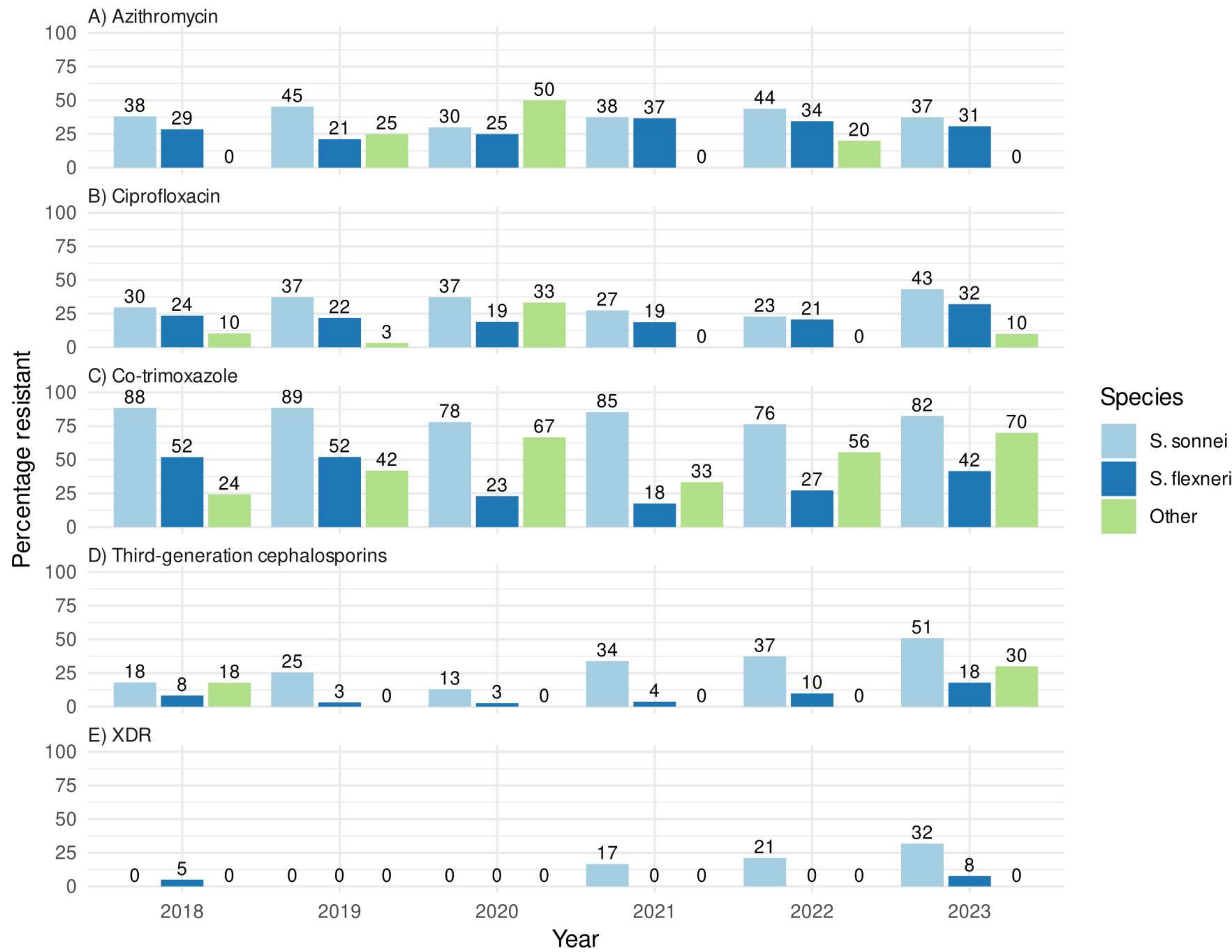

Figure S1. Percentages of isolates grouped by species phenotypically resistant against A) azithromycin; B) ciprofloxacin; C) co-trimoxazole; D) third-generation cephalosporins. Panel E) shows the percentage of isolates assessed as XDR based on phenotypic data. Only isolates with data available for all four antimicrobials were included for panel E.

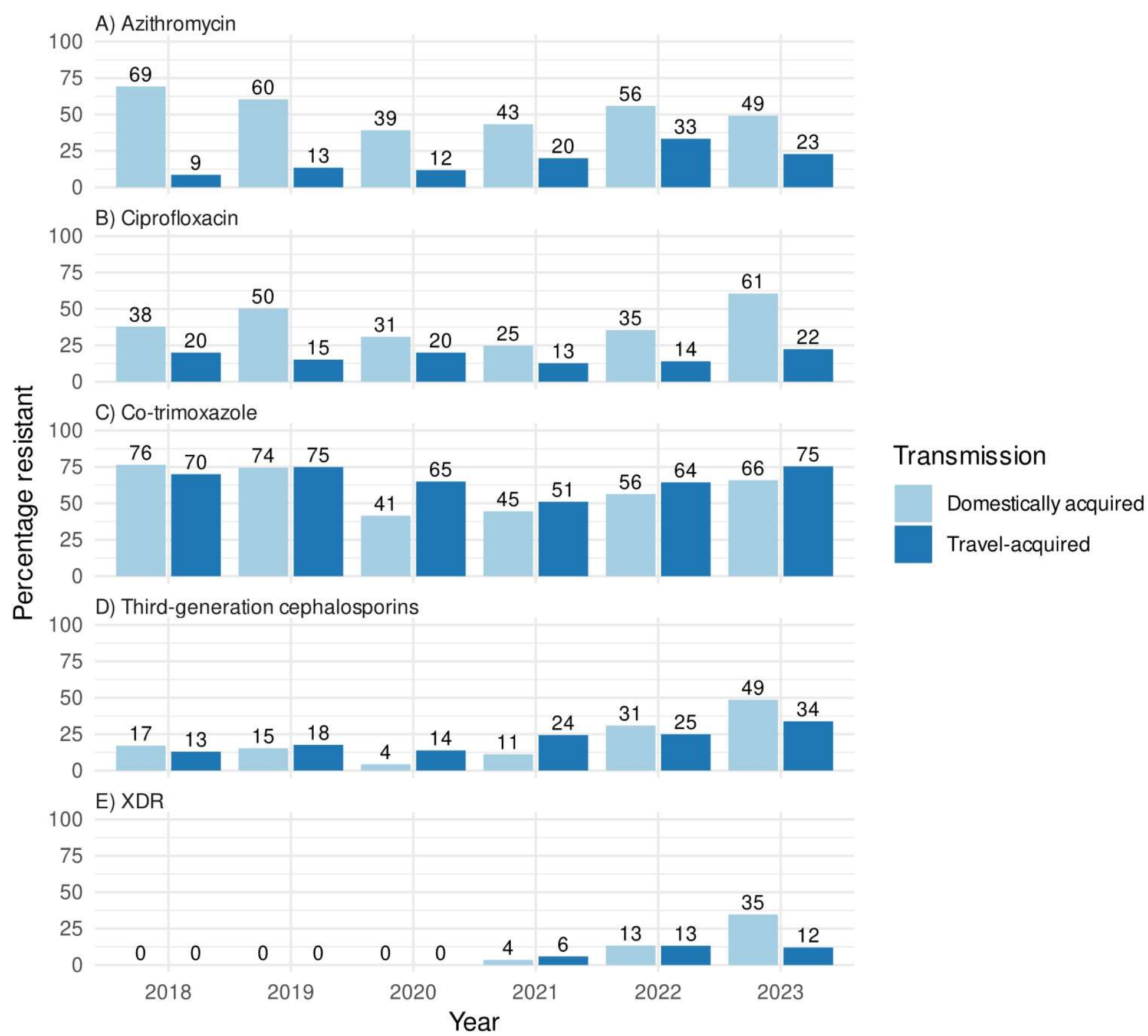

Figure S2. Percentages of isolates grouped by transmission mode phenotypically resistant against A) azithromycin; B) ciprofloxacin; C) co-trimoxazole; D) third-generation cephalosporins. Panel E) shows the percentage of isolates assessed as XDR based on phenotypic data. Only isolates with data available for all four antimicrobials were included for panel E.

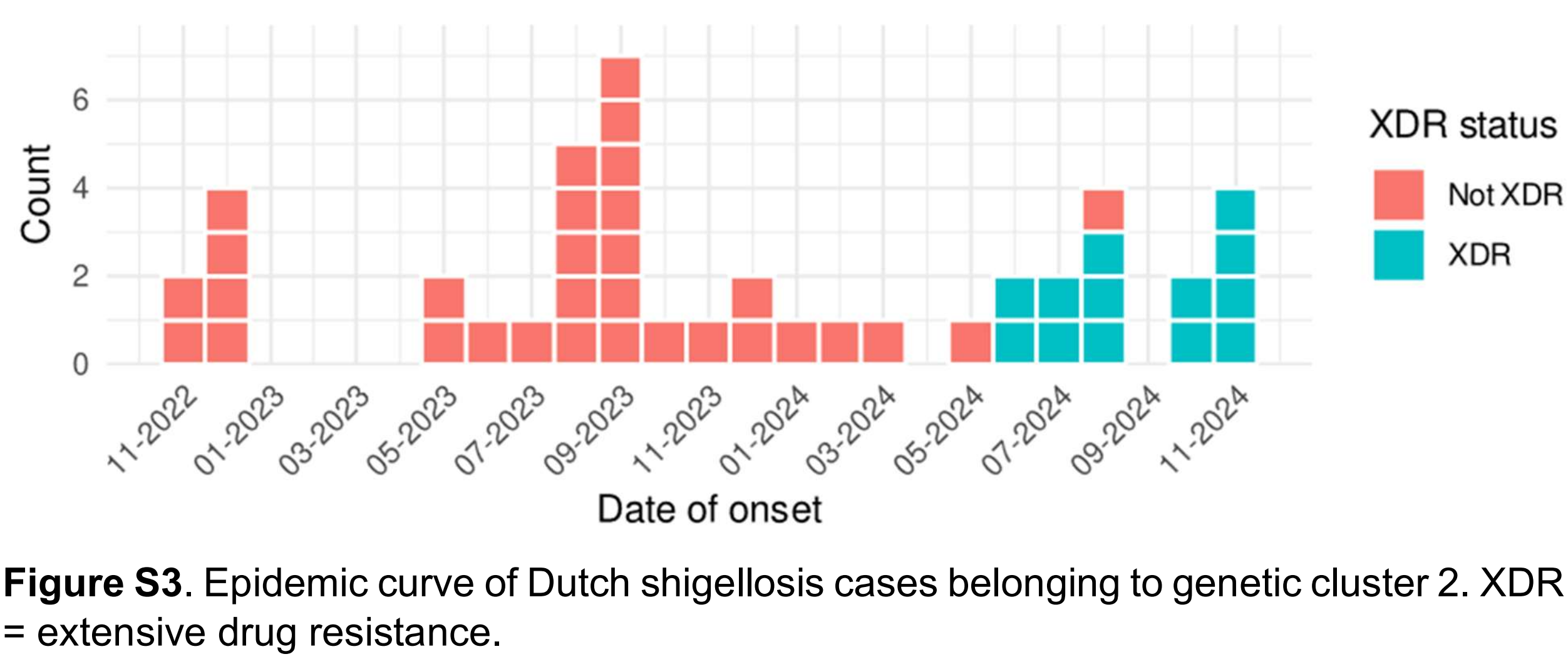

Figure S3. Epidemic curve of Dutch shigellosis cases belonging to genetic cluster 2. XDR = extensive drug resistance.
